# Massive Expansion of *Gypsy*-Like Retrotransposons in *Microbotryum* Fungi

**DOI:** 10.1093/gbe/evx011

**Published:** 2017-02-02

**Authors:** Felix Horns, Elsa Petit, Michael E. Hood

**Affiliations:** 1 Department of Biology, Amherst College, Amherst, MA

**Keywords:** transposable elements, genome defense, repeat-induced point mutation

## Abstract

Transposable elements (TEs) are selfish, autonomously replicating DNA sequences that constitute a major component of eukaryotic genomes and contribute to genome evolution through their movement and amplification. Many fungal genomes, including the anther-smut fungi in the basidiomycete genus *Microbotryum*, have genome defense mechanisms, such as repeat-induced point mutation (RIP), which hypermutate repetitive DNA and limit TE activity. Little is known about how hypermutation affects the tempo of TE activity and their sequence evolution. Here we report the identification of a massive burst-like expansion of *Gypsy*-like retrotransposons in a strain of *Microbotryum*. This TE expansion evidently occurred in the face of RIP-like hypermutation activity. By examining the fitness of individual TE insertion variants, we found that RIP-like mutations impair TE fitness and limit proliferation. Our results provide evidence for a punctuated pattern of TE expansion in a fungal genome, similar to that observed in animals and plants. While targeted hypermutation is often thought of as an effective protection against mobile element activity, our findings suggest that active TEs can persist and undergo selection while they proliferate in genomes that have RIP-like defenses.

## Introduction

Transposable elements (TEs) are selfish DNA sequences capable of self-replication within their host genomes (Doolittle and Sapienza 1980; Orgel and Crick 1980). TEs have been remarkably successful: they are found in nearly every organism studied to date (Wicker et al. 2007) and compose a large fraction of many eukaryotic genomes (Sanmiguel and Bennetzen 1998; Daboussi and Capy 2003; Bennetzen 2005; Hua-Van et al. 2005). TE activity drives the evolution of genome architecture and organismal phenotype by shuffling and duplicating genes (Lai et al. 2005; Morgante et al. 2005) and their regulatory sequences (Kunarso et al. 2010; Lynch et al. 2011; Schmidt et al. 2012; Brawand et al. 2014). In addition, TE content is a major contributor to the wide variation in genome size observed among plants, animals, and fungi (Sanmiguel and Bennetzen 1998; Kidwell 2002; Bennetzen 2005; Neumann et al. 2006; Piegu et al. 2006; Tenaillon et al. 2011; Raffaele and Kamoun 2012).

In many plant and animal genomes, TEs have proliferated in short-lived, burst-like waves of expansion that punctuate periods of population stasis (Neumann et al. 2006; Piegu et al. 2006; Pritham and Feschotte 2007; Hawkins et al. 2008; Schmidt et al. 2012; Brawand et al. 2014). Expansions of TE families may be facilitated by the disruption of genome regulatory mechanisms, such as RNA silencing and DNA methylation, which function as genome defenses by suppressing TE activity (Selker et al. 1987; Miura et al. 2001; Galagan and Selker 2004; Buchon and Vaury 2005; Cerutti and Casas-Mollano 2006; Brennecke et al. 2007; Chung et al. 2008). Despite the important role of TEs in genome evolution across kingdoms of life, the pace and tempo of TE activity in fungal genomes have not been characterized.

Many fungal genomes have a defense mechanism known as repeat-induced point mutation (RIP), which hypermutates duplicated nuclear DNA (Selker 2002; Clutterbuck 2011; Horns et al. 2012). For example, the RIP process of the ascomycete *Neurospora crassa* introduces C-to-T mutations at CpA dinucleotides mostly within linked sequences that are longer than ∼400 bp (or ∼1000 bp in the case of unlinked sequences; Watters et al. 1999) and sharing greater than ∼80% nucleotide identity (Cambareri et al. 1991). RIP mutations can regulate TE activity by causing inactivating substitutions in TE genes (Kinsey et al. 1994; Cambareri et al. 1998; Margolin et al. 1998) and inducing silencing DNA methylation (Singer et al. 1995; Lewis et al. 2009). RIP mutations also reduce sequence similarity between initially identical TE insertions, thereby reducing the rate of ectopic recombination.

Repetitive elements in basidiomycete fungi of the genus *Microbotryum* display hypermutation patterns that resemble a RIP-like defense targeting the trinucleotide TpCpG (Horns et al. 2012). Nevertheless, these genomes contain a large fraction of TEs (≥15%) from diverse families, including LTR (*Copia*-like and *Gypsy*-like) and non-LTR retrotransposons, and *Helitron* elements (Hood 2005). A RIP-like genome defense may be expected to impact TE activity, population structure, and evolution. While observations of such effects have been lacking, co-occurrence of TEs and a RIP-like hypermutation process in *Microbotryum* offers an opportunity to provide new insights.

Here, we investigated the evolutionary dynamics of *Gypsy*-like retrotransposons in *Microbotryum*, a genus known as the anther-smut fungi. This common name derives from the pathogenic life cycle of the fungi, which includes the systemic colonization of plants in the carnation family (Caryophyllaceae) and replacement of the host's pollen in the flowers with fungal spores. Insect pollinators then spread the spores from diseased to healthy plants. By sequencing *Gypsy*-like TE populations from two strains of *Microbotryum* and analyzing their sequence variation in a phylogenetic framework, we found that *Gypsy*-like TEs have undergone a recent, strain-specific expansion in a lineage of *Microbotryum*. We found evidence that RIP-like hypermutations impact the fitness of TEs, limiting their proliferation and imposing a selective sieve that favors TE insertions that evade the RIP defense.

## Materials and Methods

**Fig. 1.—:**
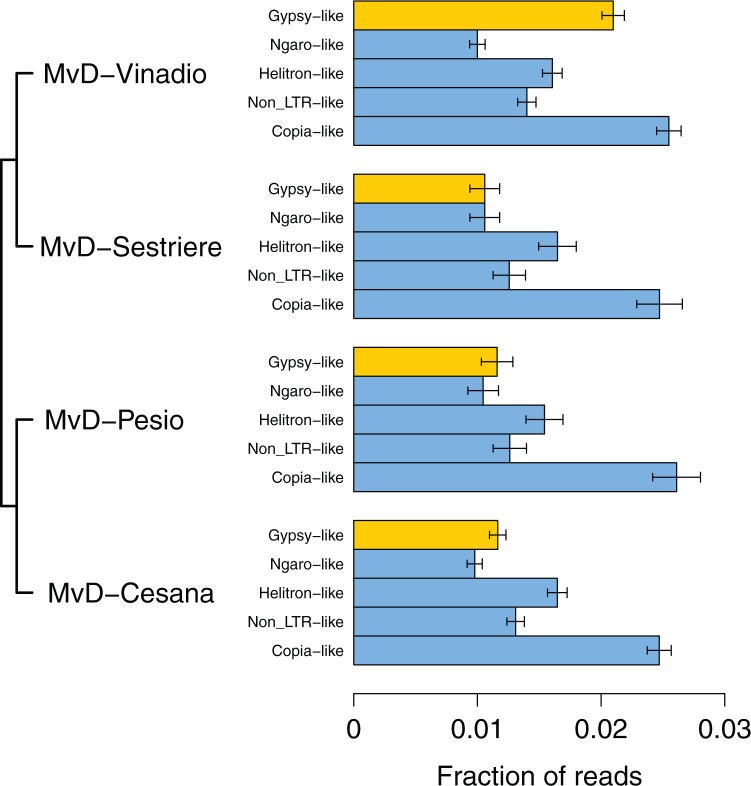
Evolutionary relationships and genomic TE content of *Microbotryum* strains. Bars display the fraction of whole genome sequencing reads aligning to repetitive element families. Error bars indicate 95% confidence intervals based on binomial sampling. Neighbor-joining phylogeny of *Microbotryum* strains was constructed based on γ-tubulin DNA sequences.

We initially assessed TE content in four field isolates of *Microbotryum* from host plants in the genus *Dianthus*, which we identify here by the abbreviation “MvD” followed by their geographic origin (table 1): MvD-Vinadio, MvD-Sestriere, MvD-Pesio, and MvD-Cesana. Previously subsumed in the epithets *Microbotryum violaceum* or *Microbotryum dianthorum*, the anther-smut fungi on *Dianthus* hosts are not yet resolved taxonomically (Le Gac et al. 2007; Denchev et al. 2009; Kemler et al. 2012). Identification of the samples used in this study and inference of the relationships among them was performed by comparing DNA sequences of the internal transcribed spacer (ITS) regions 1 and 2 of the ribosomal RNA complex (as in Freeman et al. 2002) and the intron of the γ-tubulin gene (as in Le Gac et al. 2007). Based upon γ-tubulin sequences, the named *Microbotryum* species closest to the current samples were *M. carthusianorum* for strains MvD-Vinadio and MvD-Sestriere, *M. superbum* for MvD-Pesio, and *M. shykoffianum* for MvD-Cesana (Denchev et al. 2009). The sister relationships among the four samples inferred using ribosomal ITS regions and γ-tubulin were the same (fig. 1).

**Table 1:** Origin of *Microbotryum* Samples, Showing the Host-of-Origin and Geographic Location

Strain ID	Host Species	Location	Lat., Long.
MvD-Pesio	*Dianthus pavonius*	Valle Pesio	44.196, 7.680
MvD-Cesana	*Dianthus sylvestris*	Cesana Torinese	44.937, 6.805
MvD-Sestriere	*Dianthus carthusianorum*	Colle Sestriere	44.976, 6.884
MvD-Vinadio	*Dianthus pavonius*	Sant'Anna di Vinadio	44.260, 7.123

To estimate the TE content of each genome, we sequenced the genomes using the GS-FLX platform (454 Life Sciences) with 250 bp reads covering 20–71% of each genome at ≥1X coverage. We compared all reads to the Repbase database of repetitive sequences (Jurka 2000) using tBLASTx. We estimated the genomic abundance of each TE family represented in Repbase by calculating the fraction of reads having significant similarity to that family (E-value < 10^−2^). Because mobile elements from the Pucciniomycotina are poorly represented in the Repbase database, the divergence between query sequences and target sequences in the genomes that we studied might be high. We therefore chose to use the translated BLAST search because it has high sensitivity to detect divergent sequences. This genomic shotgun sequencing approach combined with translated BLAST analysis has given predictions of relative TE content in different isolated chromosomes (Hood et al. 2004; Fontanillas et al. 2015) that were later verified by analyses of high-coverage annotated whole-genome sequencing (Perlin et al. 2015).

Next, we sampled *Gyspy*-like TEs from strains MvD-Vinadio and MvD-Sestriere using a chromosome-structured sampling approach that allowed distinguishing TE insertions with identical sequences on different chromosomes (Hood et al. 2005). We isolated DNA from individual chromosomes by separating the karyotypes of haploid meiotic products using pulsed-field gel electrophoresis. Electrophoresis was conducted on a CHEF-DRII pulsed-field system (Bio-Rad) with run conditions of 14 °C, 96 h, 2.7 V/cm, 0.8% agarose, 200 s initial and 1100 s final switch times. To separate larger chromosomes, separate electrophoresis runs were conducted with conditions of 2.1 V/cm, 2% agarose, 1100 s initial and 1800 s final switch times. We extracted chromosome-specific DNA by removing an agarose gel plug from each karyotype band using a glass Pasteur pipette. The plugs were then pulverized and twice frozen and thawed in 1X Tris-EDTA buffer. Most karyotype bands were well-resolved and therefore likely contained DNA from a single chromosome. One agarose gel plug from MvD-Vinadio came from a less well-resolved region and may have contained DNA from more than one chromosome.

We amplified by PCR, cloned, and sequenced *Gypsy*-like TE fragments from each chromosome. PCR primers were designed to amplify 938bp of the consensus of an alignment of shotgun sequence reads from *Microbotryum* strains tiled against a full-length *Gypsy*-like TE from *Laccaria bicolor* strain S238N-H82 (gi 170112043): forward primer MvGf 5′ AAAGTCCCCACGGCGTTAT and reverse primer MvGr 5′ GTTGAGAAACTGGGGTTAGCTGT. These primer sequences contain no TpCpG hypermutation target sites and flank a fragment of the *pol* gene that spans about half of the conserved reverse transcriptase domain and half of the protease domain. PCR was performed in a total volume of 10 μL containing 1 μL of chromosome-specific DNA solution, 0.25 μM forward and reverse primer, 1X PCR buffer, 1.5 mM MgCl_2_, 0.05 mM dNTP, and 0.1 units Taq polymerase (New England Biolabs). The thermal profile consisted of initial denaturation at 95°C for 3 min; 30 cycles of amplification with each comprising 95°C for 30 s, touchdown from 64°C to 59°C with a 1°C decrease after every six cycles for 30 s, and 68°C for 1 min; and final extension at 68°C for 7 min. We cloned the resulting products using a TOPO TA cloning kit (Invitrogen), then re-amplified forty clones from each chromosome using the PCR protocol described earlier, except with the initial denaturation step increased to 10 min. We purified the PCR products using ExoSAP-IT (Affymetrix). Clones were sequenced using the BigDye Terminator kit on an ABI Prism 377XL sequencer (Applied Biosystems) at the University of Massachusetts Amherst DNA sequencing facility. We excluded short sequence reads (< 700 bp) from our analysis.

We obtained 329 sequences from MvD-Vinadio and 282 from MvD-Sestriere. Because identical cloned sequences within a chromosome sample might represent amplification products from the same locus, we conservatively included in our analysis only non-redundant sequences from each chromosome, which represent distinct TE insertions. After excluding redundant sequences, 274 sequences from MvD-Vinadio and 225 from MvD-Sestriere remained, with an average of 27 unique insertions per chromosome.

To analyze sequence variation within these TE populations, we aligned the sequences using MUSCLE (Edgar 2004) and constructed maximum-likelihood phylogenies using FastTree (Price et al. 2010). Because the mutational effects of TpCpG hypermutation could obscure phylogenetic relationships among the sequences, all potential hypermutation target sites, including unmutated (TpCpG and CpGpA) and mutated (TpTpG and CpApA) sites on both strands, were excluded from the phylogenetic analysis. Phylogenies were rooted using a *Gypsy*-like TE sequence from *Microbotryum violaceum* from host plants in the genus *Silene* as an outgroup. We reconstructed ancestral sequence states by maximum-likelihood. We inferred the relative fitness of the sampled sequences based on the branching patterns of the phylogenetic trees (Neher et al. 2014). Analyses were performed using custom scripts written in Java, Python, and R, which are available upon request.

## Results

### Elevated Abundance of *Gypsy*-Like TEs in MvD-Vinadio

Using whole-genome sequencing on the 454 GS-FLX platform to assess the TE content of each *Microbotryum* strain, we found that a significantly larger fraction of reads from MvD-Vinadio align with *Gypsy*-like TEs (2.0%) compared with any of the other closely related *Microbotryum* strains (0.9–1.1%) (fig. 1; *P *= 7 × 10^−24^, Pearson’s χ^2^ test, two-sided). In contrast, all four strains carry comparable amounts of other TE families that were present in the Repbase repeat database, including *Copia*-like, *CcNgaro3-*like, non-LTR, and *Helitron*-like TEs (minimum χ^2^ comparison *P* = 0.035). This suggests that an expansion of *Gypsy*-like TEs occurred in the lineage leading to MvD-Vinadio after it diverged from the MvD-Sestriere lineage, resulting in a two-fold increase in genomic copy number.

### Recent Burst-Like Proliferation of *Gypsy*-Like TEs in MvD-Vinadio

Targeted sequencing of *Gypsy*-like TE insertions in MvD-Vinadio and its close relative MvD-Sestriere provided confirmation that *Gypsy*-like TEs have recently proliferated in MvD-Vinadio and enabled characterization of the tempo of TE activity. We sampled *Gypsy*-like TE insertions from the intragenomic populations of MvD-Vinadio and MvD-Sestriere, and sequenced a ∼1 kb fragment spanning the conserved reverse transcriptase and protease domains of the *pol* gene (Materials and Methods; Hood et al. 2005).

**Fig. 2.—:**
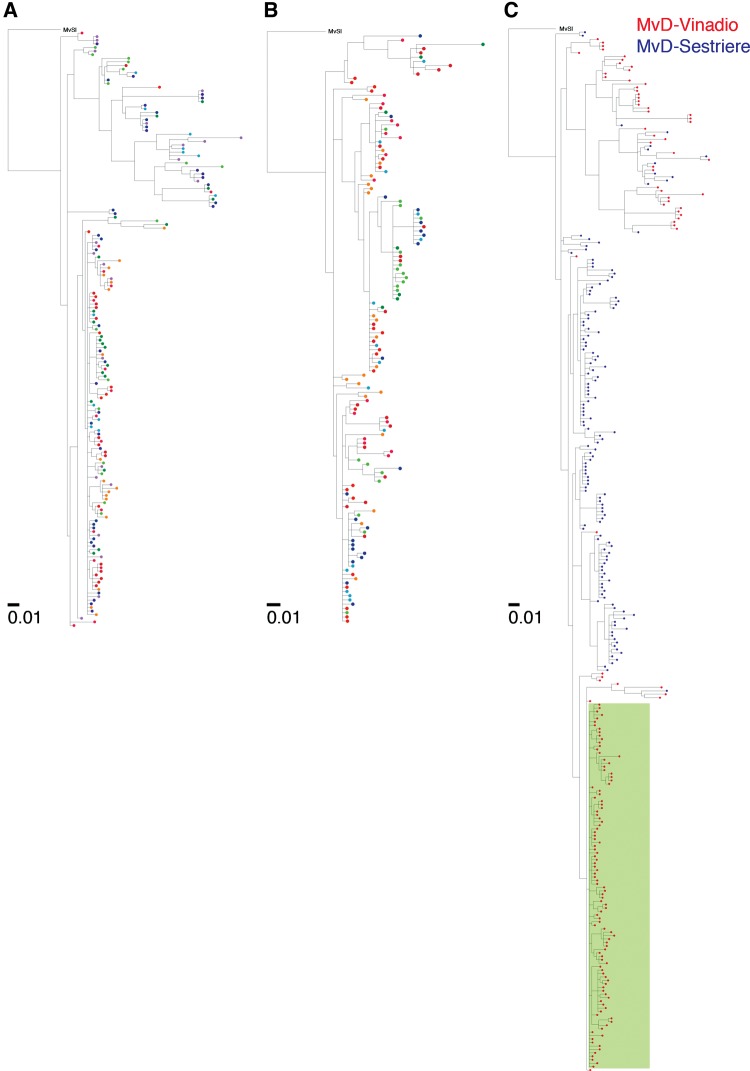
Phylogenies of *Gypsy*-like retrotransposon sequences from *Microbotryum* strains. Phylogenies of *Gypsy*-like TE sequences from (*A*) MvD-Vinadio, (*B*) MvD-Sestriere, and (*C*) both genomes were reconstructed by maximum-likelihood. In **(***A***)** and **(***B***)**, color indicates chromosome of origin. In **(***C***)**, color indicates genome of origin. Green shading in **(***C***)** indicates MvD-Vinadio-specific clade. Outgroup is a *Gypsy*-like TE sequence from *Microbotryum lychnidis-dioicae* from the host plant *Silene latifolia* (MvSl). Branch support values are indicated in supplemental figure 1 (Supplementary Material online).

Intragenomic phylogenetic analysis of *Gypsy*-like TEs, 274 from MvD-Vinadio and 225 from MvD-Sestriere, uncovered evidence of a burst-like expansion of these TEs in MvD-Vinadio. In the genome of MvD-Vinadio ∼70% of the TEs belong to a single clade of highly similar elements having mean pairwise sequence identity of 99.0% (fig. 2*A*), while a group of such highly similar TEs is not present in MvD-Sestriere (fig. 2*B*). When we reconstructed a phylogenetic tree using aligned sequences from MvD-Vinadio and MvD-Sestriere together, we found that these TEs from MvD-Vinadio form a distinct strain-specific clade (fig. 2*C*). We note that numerous sequences from MvD-Vinadio cluster with those from MvD-Sestriere elsewhere in the tree, indicating that our approach was able to detect similar TE sequence diversity in both genomes. Furthermore, older insertions shared by descent between MvD-Vinadio and MvD-Sestriere are present at similar frequency, indicating that the MvD-Vinadio-specific clade of TEs is not explained by biased sampling.

**Fig. 3.—:**
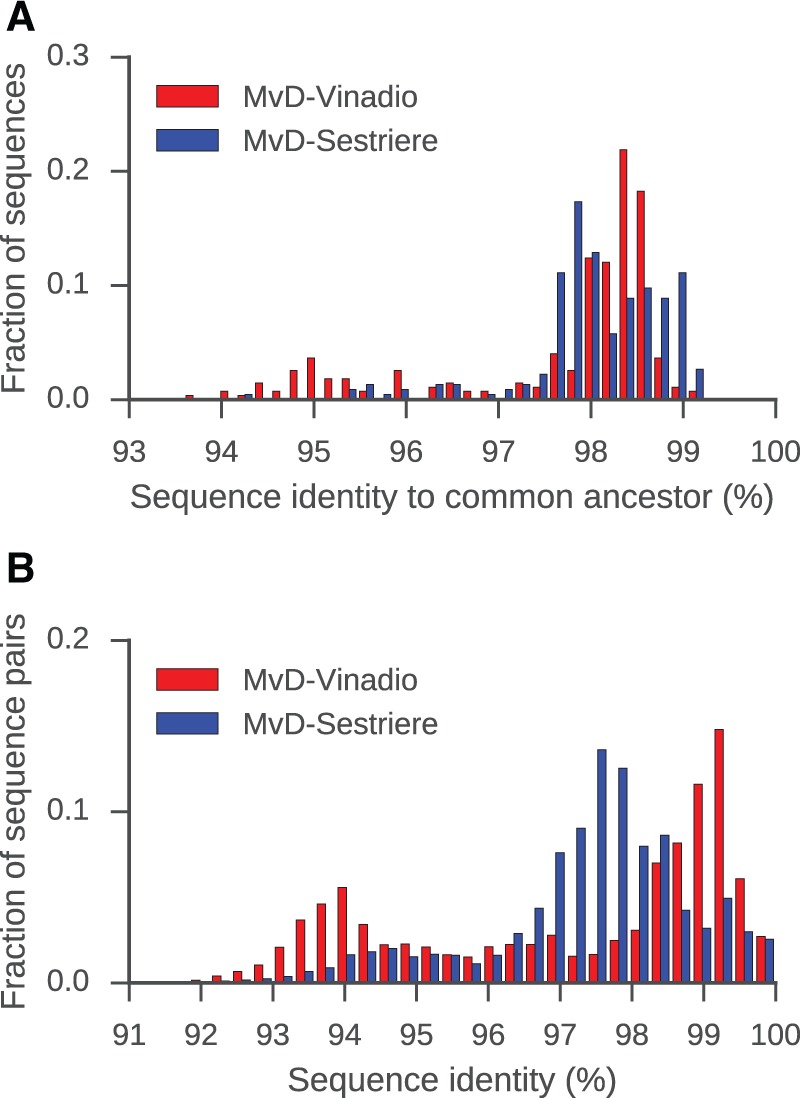
Sequence diversity of *Gypsy*-like retrotransposons from MvD-Vinadio and MvD-Sestriere. (*A*) Distributions of sequence identity to the common ancestor of all extant sequences in each genome. (*B*) Distributions of pairwise sequence identity among extant sequences in each genome.

Analysis of sequence diversity among the TE insertions in each genome supports this interpretation of a burst-like expansion of *Gypsy*-like TEs in MvD-Vinadio (fig. 3). We compared each sequence to the common ancestor of extant sequences in each genome inferred by maximum likelihood. For TEs from MvD-Vinadio, the distribution of sequence distances to their common ancestor has a sharp peak at ∼98.5% identity. This distribution is significantly different from the more balanced distribution seen for TEs from MvD-Sestriere (fig. 3*A*; *P* = 8.8 × 10^−4^; Kolmogorov–Smirnov test, two-sample). This finding suggests that most *Gypsy*-like TE insertions in MvD-Vinadio were born during a short temporal window, implying a burst-like expansion event. This conclusion is further supported by the distributions of pairwise sequence identity among extant sequences within each genome (fig. 3*B*). TEs from MvD-Vinadio exhibit higher pairwise sequence identity compared to TEs from MvD-Sestriere (*P* = 1.5 × 10^−38^; Mann-Whitney *U* test, two-sided), including a sharp peak at 99% identity composed of pairs of sequences belonging to the recent expansion. New TE insertions in MvD-Vinadio were found on all chromosomes at similar frequency (supplemental fig. 2, Supplementary Material online).

### Fitness Effects of RIP-Like Hypermutation on *Gypsy*-Like TEs

Next, we examined how the RIP-like genome defense influences *Gypsy*-like TE sequence evolution. Comparing the two focal strains, 68% of TEs from MvD-Vinadio and 55% of TEs from MvD-Sestriere display evidence of RIP-like, sequence context-dependent hypermutation (i.e., TpCpG to TpTpG transitions, or transitions in the reverse complement of this site) when compared to the common ancestor of extant elements. Each TE copy contains 1.7 ± 2.1 mutated TpCpG target sites (mean ± s.d.) among the 35 target sites present in the ancestral sequence. Mutation rates in TpCpG target sites were elevated compared to overall mutation rates of trinucleotide contexts (data not shown; see Horns et al. 2012).

**Fig. 4.—:**
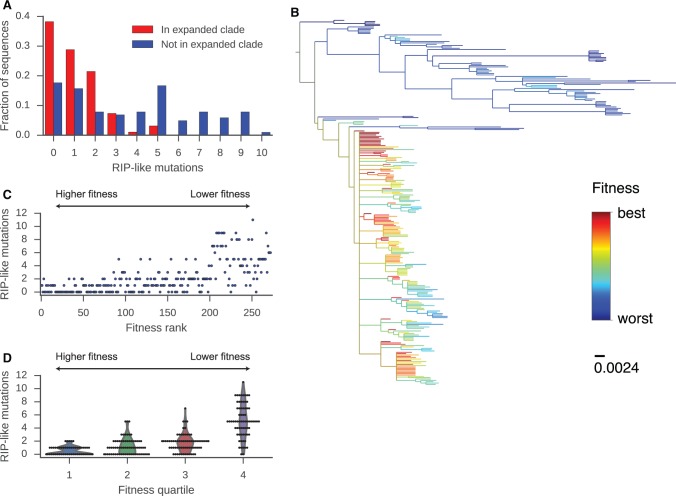
Analysis of RIP-like mutations in *Gypsy*-like retrotransposons. (*A*) Distributions of the number of RIP-like mutations in sequences either belonging or not belonging to the strain-specific expansion in MvD-Vinadio. **(***B***)** Maximum-likelihood phylogeny of *Gypsy*-like TEs in MvD-Vinadio colored by inferred relative fitness based on local branching index. **(***C***)** Number of RIP-like mutations in extant sequences from MvD-Vinadio plotted against relative fitness rank (rank closer to 0 is higher fitness). **(***D***)** Number of RIP-like mutations in extant sequences in each quartile of relative fitness (quartile 1 is highest fitness). RIP-like mutations were determined by comparison with the ancestor of all extant sequences inferred by maximum-likelihood .

By assessing mutation patterns of *Gypsy*-like TEs involved in the recent expansion in MvD-Vinadio, we found evidence that RIP-like mutations suppress TE activity. Strikingly, we found that TEs belonging to the recently expanded clade (*N* = 191 sequences) have fewer RIP-like mutations than other TEs (fig. 4*A*; *P* = 1.8 × 10^−13^; Mann-Whitney *U* test, two-sided). The median number of RIP-like mutations is 1 among TEs within the expanded clade, while the median is 4 among TEs outside the expanded clade. The DNA sequence of the ancestral TE that initiated the expansion in MvD-Vinadio, reconstructed by maximum likelihood, contained no mutations of TpCpG site in comparison with the common ancestor of extant elements. These findings suggest that evasion of RIP-like hypermutations was associated with TE proliferation. That some of the sequences within the expanded clade contain TpCpG site mutations suggests that the putative RIP-like genome defense was active during or after the expansion.

To examine how the RIP-like genome defense affects the fitness of *Gypsy*-like TEs in MvD-Vinadio, we inferred the relative fitness of each extant TE sequence based on the branching patterns of the phylogenetic tree (fig. 4*B*; Neher et al. 2014). This inference method is based on the notion that an individual with high fitness is expected to be found at the root of a rapidly branching clade. Similarly, extant individuals which are close descendants of these internal nodes are expected to have high fitness. Many *Gypsy*-like TEs in the expanded clade have high inferred fitness, reflecting the recent rapid proliferation of that clade. We found that the number of RIP-like mutations in extant TE sequences correlates significantly with their inferred fitness (fig. 4*C*; Spearman rank correlation coefficient 0.69; *P* = 2.0 × 10^−39^). When analyzed in quartiles, sequences in the top (first) quartile of ranked fitness contain TEs in the expanded clade and have significantly fewer RIP-like mutations compared to every other quartile (fig. 4*D*; *P* < 8.3 × 10^−5^; Mann-Whitney *U* test, two-sided). Conversely, TE sequences with more RIP mutations tend to have lower fitness. These findings suggest that *Gypsy*-like TEs which have not suffered RIP-like mutations proliferate more than TEs which have suffered such mutations. Notably, the second and third quartiles of inferred fitness also contained some TEs belonging to the expanded clade, and these had significantly more RIP-like mutations than the first quartile. Thus, even within the recent expansion, TEs that suffered more RIP-like mutations tended to proliferate less, suggesting that the RIP-like genome defense was actively limiting TE activity during the expansion, rather than only mutating sequences afterward.

## Discussion

Despite the ubiquity of TEs and their important role in shaping genome evolution, their population and evolutionary dynamics remain poorly understood. By reconstructing the evolutionary history of *Gypsy*-like TEs within closely related genomes of *Microbotryum*, we have found evidence that a lineage-specific burst of *Gypsy*-like TE proliferation has dramatically remodeled the genome composition of the strain MvD-Vinadio, doubling the genomic representation of this TE family. In addition, our results show how a RIP-like hypermutation process shapes the evolution of *Gypsy*-like TEs and suggest that hypermutation imposes a selective sieve on TE insertions.

Punctuated patterns of retrotransposon proliferation have been described in plant (Neumann et al. 2006; Piegu et al. 2006; Hawkins et al. 2008) and mammalian genomes (Pritham and Feschotte 2007; Lynch et al. 2011; Schmidt et al. 2012). In this work, we have presented evidence for a massive burst-like expansion of retrotransposons in a fungal genome. While sequence differences due to PCR errors may be present in our analysis, this source of error would not be expected to affect the genomes differently nor to create the patterns consistent with proliferation that we observed. The narrow peak width in the distributions of sequence diversity among *Gypsy*-like TEs in MvD-Vinadio suggests that their proliferation occurred during a temporally compressed period. The proliferated TEs became dominant within the intragenomic population, indicating that this brief episode of proliferation has strongly driven the evolution of *Gypsy*-like TEs in this genome. While the causes underlying this episode of transposition activity remain undetermined, it is notable that the genus of *Microbotryum* has a history of hybridization (Devier et al. 2010). Hybridization (Bingham et al. 1982; Liu and Wendel 2000; Ungerer et al. 2006) or environmental stress (McClintock 1984) might trigger TE proliferation by compromising regulatory mechanisms such as DNA methylation or RIP.

Patterns of RIP-like hypermutation with a trinucleotide target preference of TpCpG are conserved among fungi of the Pucciniomycotina subphylum of the Basidiomycota (Horns et al. 2012). While caution is necessary in the absence of direct evidence for a RIP pathway, such as may be provided by transformation experiments (Toh et al. 2016), our data provide further evidence that a RIP-like hypermutation process targeting TpCpG has affected TE evolution in *Microbotryum*. Remarkably, the observed expansion of *Gypsy*-like TEs appears to have occurred in the face of concurrent activity of a RIP-like hypermutation process. Our results demonstrate that RIP-like mutations impact TE fitness by limiting the proliferation of individual TE insertions. This suggests that evading a RIP-like defense may be key for the success of retrotransposons in genome colonization. Selection may favor TE variants that are able to evade hypermutation, including variants that have shed RIP target sites. Proliferation of TE variants that evade RIP-like mutations exposes the host genome to elevated mutational load and risk of ectopic recombination. These costs in turn exert selective pressure on the host genome to curtail evasion and suppress TE proliferation, highlighting the potential of TE activity to drive an intragenomic arms race.

In other fungal species, RIP is thought of as a remarkably effective defense mechanism against selfish DNA, including TEs. Much of the evidence for this notion comes from studies of the ascomycete fungus *Neurospora crassa* and its close relatives, which have a well-defined RIP process that targets dinucleotide sequence motifs. Unlike *Microbotryum*, the genome of *N. crassa* harbors no intact mobile elements and contains numerous fossils of mobile elements that have evidently suffered RIP mutations (Galagan et al. 2003). Our results suggest that genomes having a RIP-like hypermutation process may be more permissive to the maintenance of TE activity than previously thought. We propose that the less frequently occurring trinucleotide target site sequence of the RIP-like hypermutation process in *Microbotryum* may render this defense less effective at suppressing TE activity than the dinucleotide-targeting RIP process found in *Neurospora*. Mechanisms that influence the pace and tempo of TE activity and permit coexistence of TEs and genome defenses deserve further investigation.

## Supplementary Material

Supplementary Data
