# Supplementary figures and images for: Massive Expansion of Gypsy-Like Retrotransposons in Microbotryum Fungi
Source: Genome Biol Evol. 2017 Feb 2;9(2):363–71. doi: 10.1093/gbe/evx011 (PMC5381629; doi:10.1093/gbe/evx011)

**A**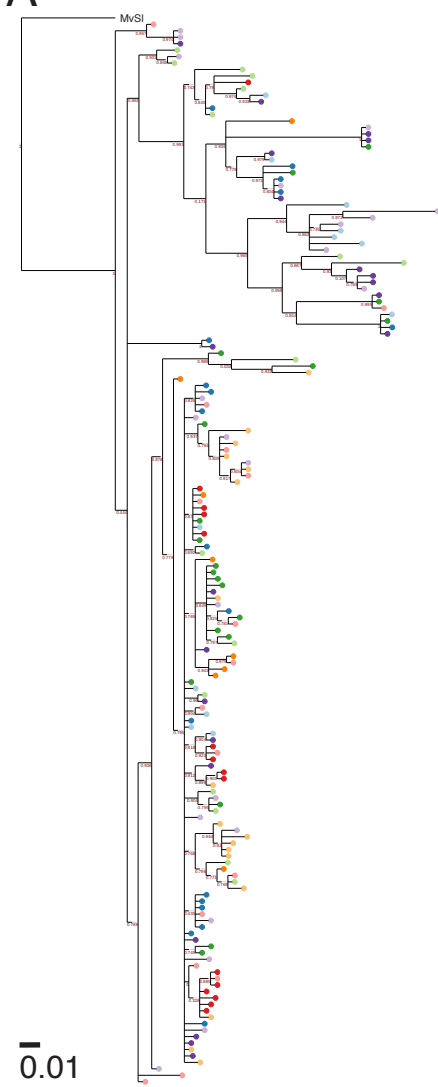**B**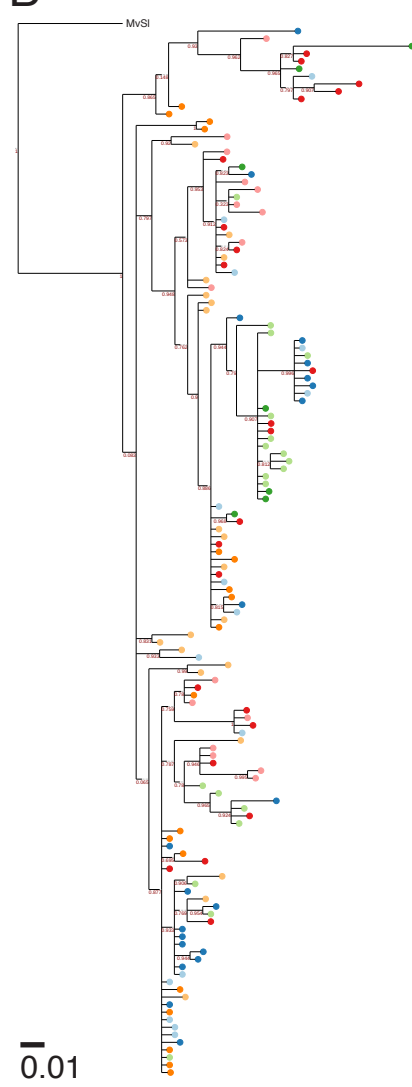**C**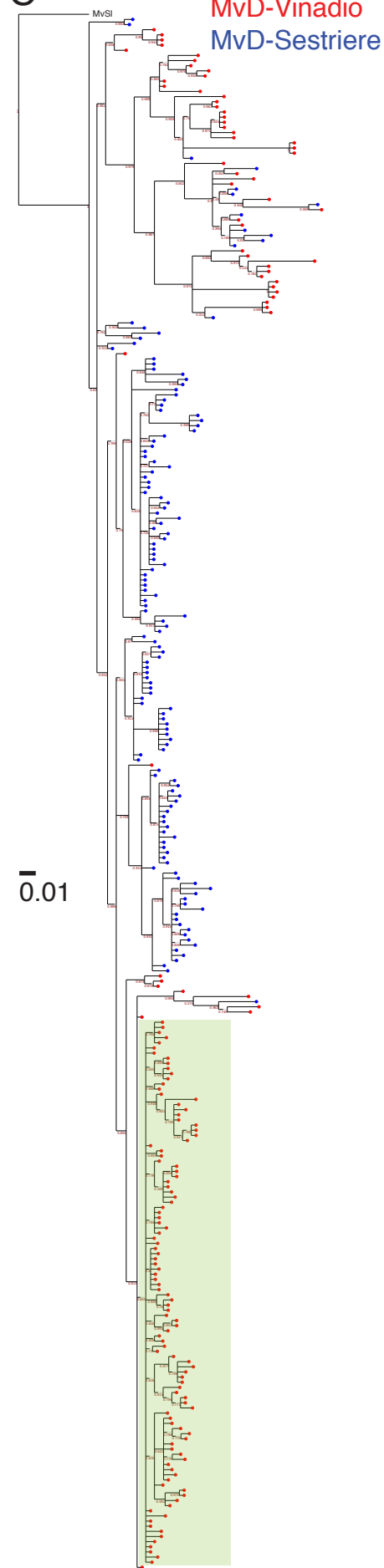

Supplement: Supplementary Data [file evx011_Supp.zip › SuppFig1.pdf]

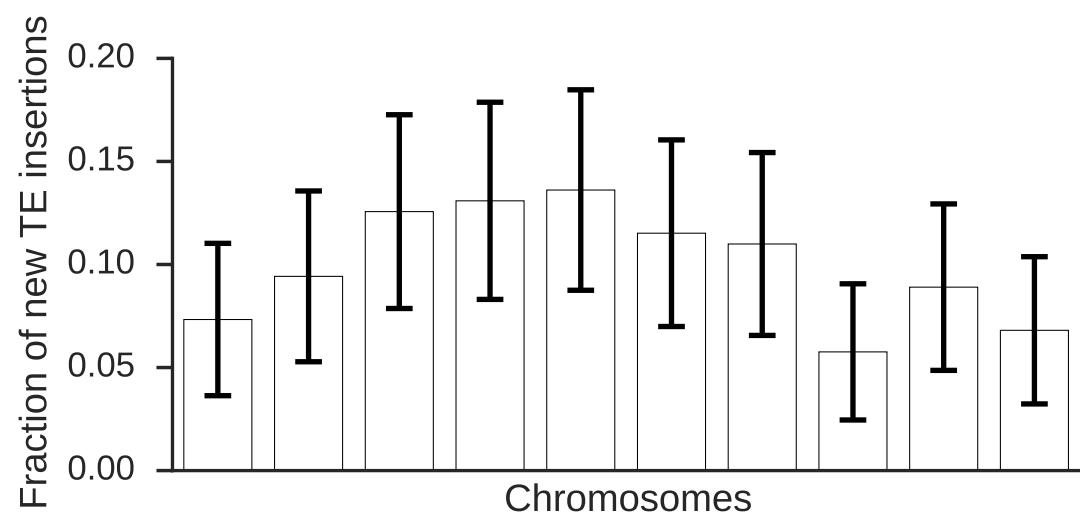

Supplement: Supplementary Data [file evx011_Supp.zip › SuppFig2.pdf]
